# High-Resolution Optical Fiber Temperature Sensor Based on Draw Tower Grating Array

**DOI:** 10.3390/s22082846

**Published:** 2022-04-07

**Authors:** Hanjie Liu, Ciming Zhou, Yandong Pang, Xi Chen, Ye Xu, Dian Fan

**Affiliations:** 1 School of Information Engineering, Wuhan University of Technology, Wuhan 430070, China; liuxiaoniu@whut.edu.cn (H.L.); zcm@whut.edu.cn (C.Z.); cx2016@whut.edu.cn (X.C.); xyeee@whut.edu.cn (Y.X.); 2 National Engineering Research Center of Fiber Optic Sensing Technology and Networks, Wuhan 430070, China; 3 School of Military Engineering, Naval Engineering University, Wuhan 430070, China; pydgogogo@whut.edu.cn

**Keywords:** oceanography, temperature measurement, interferometers, fiber optical sensors, phase modulation

## Abstract

Ocean temperature monitoring is of great significance to marine fishing, aquaculture, and marine operations. Traditional electric sensors lack the potential to multiplex several sensors, and may suffer from electromagnetic interference. Meanwhile, fiber Bragg grating-based sensors have the advantages of high sensitivity, possibility for large-scale multiplexing, and immunity to electromagnetic interference. In this paper, we propose a Fabry–Pérot (FP) interferometer based on the draw tower grating array and combine it with the phase measurement method for demonstration and testing. In the sensor system, two adjacent fiber Bragg gratings (FBGs) are used as mirrors and an optical fiber connects them, forming a sensor unit. The signal was detected through the compensation of the optical path difference via two-arm path differences in an unbalanced interferometer. The sensor is calibrated in the range of 36.00–36.50 °C, and back to 36.00 °C, in steps of 0.10 °C. A thermocouple (DW1222) is used as a reference. Experimental testing demonstrates that under the thermal loop, the temperature and phase can be approximated as a linear relationship, the Pearson square correlation coefficient is 0.9996, and the temperature sensitivity is −9846 rad/°C. To prove that our experimental device can achieve a higher temperature resolution, we measured the background noise of the system. The experimental results indicate that the order of magnitude of our system temperature resolution can reach 10^−5^ °C. Thus, we believe that the sensor system is promising for the application of ocean temperature detection, and owing to the ultraweak reflection characteristics of the FBG, this method provides the possibility for large-scale multiplexing of the system.

## 1. Introduction

In situ monitoring of temperature parameters is important for process control in manufacturing businesses, protection of ecosystems, and prevention of global warming, especially in real-time monitoring of the ocean temperature to prevent the occurrence of marine natural disasters. Traditionally, thermal resistance and thermocouple are the earliest developed and most widely used temperature sensors [1,2]. With the development of semiconductor technology, researchers have invented a semiconductor thermocouple sensor [3], a PN junction temperature sensor [4], and an integrated temperature sensor [5]. Although electronic-type sensors have the advantage of high precision and wide-ranging practicality, the sensors lack the potential to multiplex several sensors, and the complex environment of the ocean accelerates the consumption of the sensor’s electrical energy.

In recent years, fiber-optic sensors have received considerable attention for their unique advantages such as possibility for large-scale multiplexing [6], immunity to electromagnetic interference [7], and they can operate without electrical powering or local batteries outside the terminal nodes [8], especially the weak grating interferometric fiber-optic sensor based on the draw tower grating array. Due to the extremely low reflectivity of its grating mirrors, which minimizes high-order crosstalk, the technology can be applied in various fields to meet the needs of large-scale multiplexing and high sensitivity measurement [9]. In 1993 in Germany, Dong et al. [10] first proposed the technology of writing grating during fiber drawing, and wrote gratings in the photosensitive fiber drawing process by single-pulse holographic interference method. In 1995, Askins et al. [11] improved the method to write gratings of different wavelengths online. In 2011, German researchers completed the preparation of commercial draw tower gratings [12]. In 2015, Wang et al. [13] conducted an underwater acoustic signal demodulation experiment using a 500-element draw tower grating array. The system can demodulate underwater sound pressure signals with a minimum of 0.122 Pa, and obtain sound pressure sensitivity of −158 dB under the stimulation of 450–600 Hz sound waves. In 2019, Gan W. et al. [14] verified that the ultra-weak FBG array can meet the technical requirements of distributed dynamic measurement in practical engineering. In 2020, Xin L. [15] et al. proposed a method to identify surface intrusion events using ultra-weak fiber Bragg grating array detection signals in subway tunnels. Experiments show that the scheme can identify four common events, and the average recognition rate reaches 96.57%, which can meet the needs of practical applications. However, there is no relevant report on the combination of draw tower grating and phase modulation interferometric optical fiber temperature sensors. Phase modulation fiber-optic sensors mainly use the principle of optical interference to complete signal detection, which is one of the most sensitive detection technologies in optics [16]. The geometry of the sensor probe can be designed into different forms according to requirements, which is suitable for different test environments. Therefore, we first propose a dual-beam interference structure of FP cavity based on the draw tower array, which adopts the phase measurement method to achieve high-temperature resolution.

In 1982, the method of demodulating optical fiber interference sensor using a 3 × 3 coupler was mainly proposed by the Naval Research Laboratory [17]. The demodulation scheme relies on the three outputs of the 3 × 3 coupler and uses all three of its phase-modulated output signals to reproduce the stimulus that produced the original optical phase modulation. Because the demodulation process of the algorithm involves operations such as integration, differentiation, and derivation that increase system noise, the use of this algorithm is not widespread at present. In 2006, Wang et al. [18] discussed the arctangent method of a PGC structure for optical fiber interferometer array and carried out a simulation. Compared with the 3 × 3 coupler demodulation algorithm, the arctangent demodulation algorithm not only eliminates the DC component in the interference signal, but also cancels the optical path noise and light source noise in the signal with a simple calculation process. Therefore, we used the arctangent method of a 3 × 3 structure fiber interferometer array to demodulate the phase.

In our sensing system, two adjacent fiber Bragg gratings (FBGs) are used as mirrors and an optical fiber connects them to form a sensor unit; the single beam of the lead-in sensor unit is divided into reference light and probe light. The optical path difference between the two arms of the unbalanced Michelson interferometer is used to compensate for the optical path difference between the reference and probe lights to detect the signal. In order to avoid the phase noise caused by the light source [19], we adopt a narrow linewidth fiber laser and apply the time division multiplexing method to the structure. This structure realizes that multiple adjacent sensing units can match an interferometer, which has a large-scale multiplexing capacity and simple implementation. Through the theoretical analysis of the total phase change and sensitivity composition of the FP interferometric fiber-optic temperature sensor, we use the 3 × 3 arctangent demodulation algorithm to process the interference signal and then perform a simulation to quantitatively verify the theoretical analysis. Based on the experimental results using our innovative approaches and techniques, we conclude that the temperature resolution measurement of 0.10 °C was achieved, which meets the demand of ocean day/night temperature difference measurement. By analyzing the background noise of the system, it is verified that our experimental device can achieve a high-resolution temperature measurement of the order of 10^−5^ °C, which can realize the temperature measurement of the ocean thermostatic layer. Thus, optical fiber thermometers may reach the performance and capabilities of thermocouples and be a real alternative for high-resolution temperature sensing and monitoring of the ocean environment. The practicality of the sensor provides great potential for its application in ocean temperature sensing.

## 2. Theory and Sensing System Design

### 2.1. Sensor Fabrication and Experimental Setup

**Figure 1:**
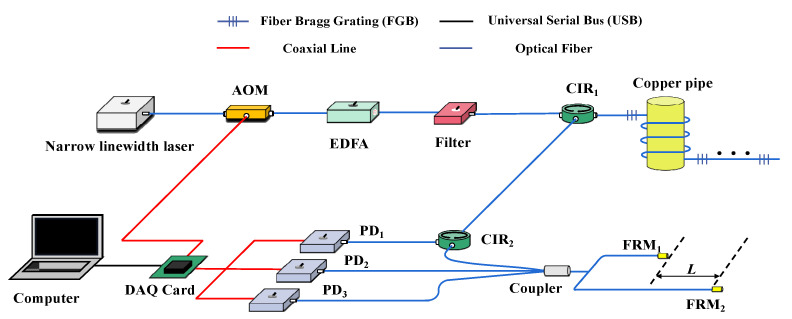
Fiber-optic temperature sensor array based on FBG.

**Figure 2:**
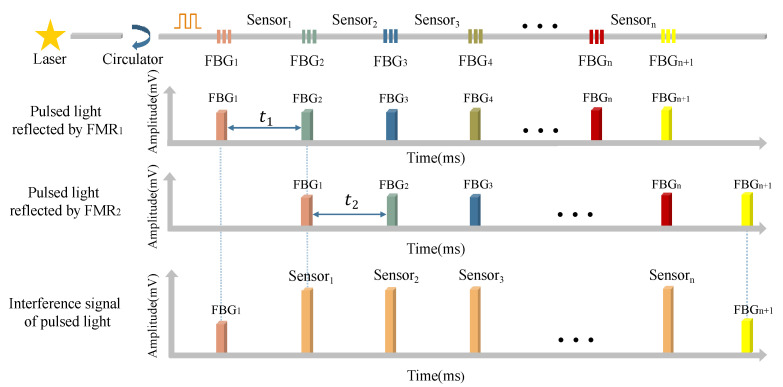
Interference in FBG array.

The experimental setup is presented in Figure 1. The narrow linewidth, which distributes a feedback laser whose center wavelength is 1550 nm, is used as a light source to output continuous light. The signal generator sends out an electric pulse signal with a fixed pulse width and repetition frequency to connect to the drive of the acousto-optic modulator (AOM), so that the continuous light output by the light source is modulated into a light pulse sequence. In the experiment, the AOM continuously sends two optical pulses; the time required for the latter optical pulse should be greater than the time required for the previous optical pulse to travel through the entire fiber grating array to be tested and return to the 3-port of the first circulator, so as to ensure that the interference signals will not overlap. Therefore, we set the repetition frequency of AOM to 20 Hz and the pulse width to 20 ns. The optical pulse generated by the AOM modulation is amplified using a fixed-gain erbium-doped fiber amplifier (EDFA). As the EDFA amplifies both the pulse and noise signals, the input end of a filter with a center wavelength of 1550 nm is connected to the output end of the EDFA to filter the amplified noise signal. The optical pulse signal output from the output end of the filter passes through the 1-port of the circulator1 (CIR1) and enters the FBG array from the 2-port. The center wavelength of the FBG is 1550 nm, and the 3 dB bandwidth is 1.5 nm. In the sensor array reported here, two adjacent FBGs are used as mirrors and an optical fiber connects them, also known as the length of the cavity, to form a sensor unit. The sensor was tightly wound on a copper tube [20] and placed in the thermostat (RTS). The fluctuation of the temperature field of the thermostat RTS is ±0.001–0.01 °C. The light pulse reflected by the FBG array enters the unbalanced Michelson interferometer through the 3-port of the CIR1. In the interference light path, the unbalanced Michelson interferometer achieves the function of a wedge plate so that the light waves meet to form interference fringes. Corresponding to the interference model in the draw tower grating in Figure 2, after the pulsed light is reflected by FBG1 and FBG2, two pulse trains are generated using the unbalanced Michelson interferometer. Owing to the optical path difference, a time delay τ occurs in the front and rear pulse trains, so that the pulse from FBG2 reflected by Faraday rotating mirror1 (FRM1) matches the optical path of the pulse from FBG1 reflected by FRM2. Then, the interference fringes of sensor 1 are formed to conduct temperature sensing. After that, three photodetectors (PDs) convert the interference signals into electrical signals. These electrical signals are collected by the high-speed data acquisition card and uploaded to the upper computer in real time for data processing and display.

The optical pulse signal obtained by AOM modulation in Figure 1 passes through port 1 of CIR1 and enters the draw tower grating array from port 2. Assuming that the array has n FBGs, there are n reflected optical pulses output from port 3 of CIR1, and the reflected optical pulse of the *i*-th FBG can be expressed as

(1) $$E_{i}(t)=E_{i}exp(2\pi \nu t)$$

where ν is the optical frequency of the light source. The reflected optical pulse of the *i* + 1-th FBG can be expressed as

(2) $$E_{i+1}(t)=E_{i+1}exp[2\pi \nu (t+t_{1})]$$

where $t_{1}=nl/c$ is the time interval between optical pulses reflected by two adjacent FBGs, and l is the distance between two adjacent FBGs. When the reflected optical pulse passes through CIR2 and enters the unbalanced Michelson interferometer composed of a 3 × 3 coupler and FRM, the reflected optical pulse of the n-th FBG passes through the long arm of the interferometer and is reflected by the FRM to the 3 × 3 coupler, which can be expressed as

(3) $$E_{i}^{\prime }(t)=E_{i}exp[2\pi \nu (t+t_{2})]$$

where $t_{2}=nL/c$ is the time interval between optical pulses reflected by two adjacent FBGs, and L is the length difference between two arms of unbalance interferometer. The reflected optical pulse of the *n* + 1-th FBG passes through the short arm of the interferometer and is reflected by the FRM to the 3 × 3 coupler, which can be expressed as

(4) $$E_{i+1}^{\prime }(t)=E_{i+1}exp[2\pi \nu (t+t_{1})]$$

Thus, it can be concluded that when the distance between two adjacent FBGs is equal to the difference in arm length of the interferometer, $t_{1}=t_{2}$. The optical pulse reflected back by the FRM of the *i*-th FBG through the long arm of the Michelson interferometer and the optical pulse of the *i* + 1-th FBG reflected back by the FRM through the short arm of the Michelson interferometer can reach the 3 × 3 coupler at the same time, resulting in the interference phenomenon. The timing diagram of the interference signal is shown in Figure 2.

There are many advantages of the design of the interference optical path structure matched with the sensor array. On the one hand, the dual-beam common optical path design greatly reduces the complexity of the system, so we need not consider the desensitization design of the reference arm. On the other hand, because the experimental setup has only the inconsistency of the light wave phase in the sensing part, the design reduces the noise of the system and the impact of polarization changes. Simultaneously, based on the adjacent grating collocation, each element of the sensor array can share a matching interferometer. This condition belongs to an interference sensing scheme that can simply realize a large-scale multiplexing.

### 2.2. Optical Interference Principle

For temperature applications, the sensing optical fiber of the FP interference cavity is affected by the temperature of the external physical quantity to be measured. A change in the refractive index, diameter of the optical fiber core, and length of the optical fiber in the sensing area induce a phase difference between the light beams traveling in both arms. Thus, the phase of the light wave transmitted through the optical fiber is changed, and it can be expressed as [21]

(5) $$\Delta \varphi =\beta \Delta L+L\left(\frac{\partial \beta }{\partial n}\right)\Delta n+L\left(\frac{\partial \beta }{\partial D}\right)\Delta D$$

where Δφ is the change in the total phase caused by environmental changes, L is the length of the sensing cavity, ΔL is the change in the length of the sensing cavity, n is the refractive index of the optical fiber, Δn is the change in the refractive index of the optical fiber, D is the diameter of the optical fiber, ΔD is the change in fiber diameter, β is the propagation constant of the optical fiber, $\beta \approx nk_{0}$, $k_{0}=2\pi /\lambda_{0}$, and $\lambda_{0}$ is the wavelength of the light output by laser. The first term in Equation (1) is the phase change caused by the change in cavity length, the second term is the phase change caused by the change in fiber refractive index, and the third term is the phase change caused by the change in fiber diameter. The last term’s phase shift is two to three orders of magnitude smaller than that of the first two terms, and therefore, it can be ignored [22]. Equation (5) can explain that interference phase changes are mainly related to changes in the refractive index and fiber length.

The optical fiber is placed in the temperature field, and the temperature change affects the change parameters n and L at the same time. Owing to the influence of the temperature, the phase change in the optical fiber can be expressed as

(6) $$\frac{\Delta \varphi }{\Delta T\cdot L}=2k_{0}\left(\frac{dn}{dT}+\frac{n}{L}\frac{dL}{dT}\right)$$

Therefore, according to Equation (6), by monitoring the phase changes, we can determine the temperature change around the sensor unit.

In the temperature sensing system, as the round-trip optical path needs to be considered, the optical path difference of the light reflected by the pair of gratings is $2k_{0}nL$, the fiber length L of the sensing element is 50 m, and the wavelength of the light output by laser is 1550 nm. In addition, the refractive index of silica glass fiber core is 1.458, the thermal expansion coefficient of copper is $\frac{1}{L}\frac{dL}{dT}=14.8\times 10^{-6}/℃$, and the sensitivity of the refractive index to temperature is $\frac{dn}{dT}=6.8\times 10^{-6}/℃$. The linear relationship between the phase change of the sensing fiber and the temperature change can be obtained by putting the aforementioned value into Equation (6):

(7) $$\frac{\Delta \varphi }{\Delta T}=11498\mathrm{rad}/℃$$

This condition indicates that a temperature change of 1 °C changes the phase of the interference signal in the sensing fiber by 11,498 rad.

### 2.3. Simulation

**Figure 3:**
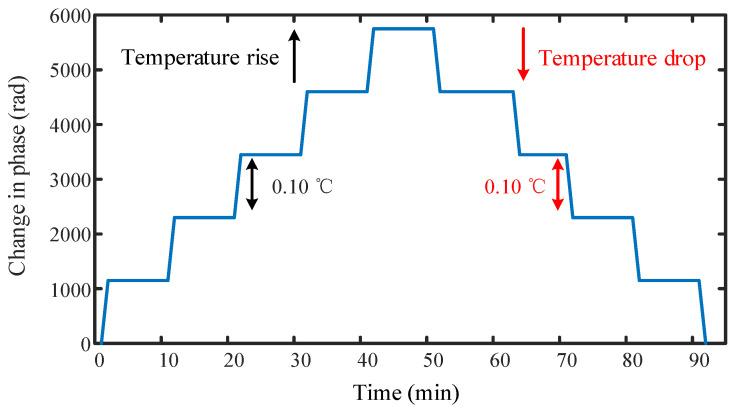
Signal reconstruction in interference.

We used the 3 × 3 arctangent algorithm [23] to demodulate the signal, and simulated the demodulation results with temperature resolutions of 0.10 °C on MATLAB. The simulation result shows a thermal loop in Figure 3. The simulation result is shown in Figure 3, where the black line is the phase change as the temperature rises, the phase changes 1149.8 rad each time the temperature rises by 0.10 °C, and the blue line is the phase change as the temperature drops, which is symmetrical with the curve as the temperature rises.

## 3. Experimental Results and Discussion

**Figure 4:**
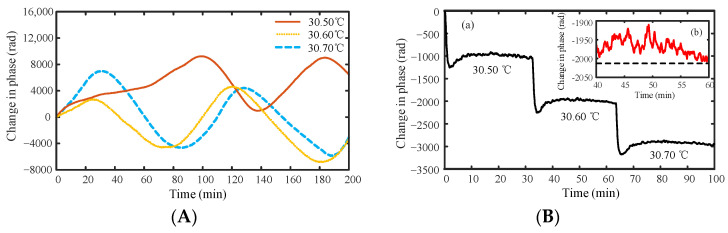
Long-term continuous measurement results of phase at a constant temperature of 30.50 °C, 30.60 °C, and 30.70 °C. (**A**) The unbalanced interference arm was exposed to air. (**B**) The unbalanced interference arm was at a constant temperature.

To verify the performance of the temperature sensor based on the draw tower grating array, we set the distance between two adjacent gratings in the sensing unit to be 50 m, and we wound them tightly around a copper pipe with a 6 cm diameter and 15 cm height. As the sensitive unit in this system was a long cavity between gratings, we used a narrow linewidth laser to enable the front and rear light to have interference effects. The linewidth coherence length must be greater than the sensing cavity length. To ensure suitable interference results, the continuous light emitted by the laser must be modulated into pulsed light via AOM. We performed tests using a high-precision automatic measurement and verification constant temperature water tank (RTS-0515) with a working tank opening of 235 × 180 mm^2^ and a depth of 200 mm. We used the PXle-1071 series (NI, Austin, TX, USA) as the data acquisition card. This card consists of four channels, of which three were acquisition channels used to detect the interferometric electrical signals of the three PDs. We used the 3 × 3 demodulation algorithm to obtain the phase change in the interference signal. This phase change is in turn used to demodulate the change in the temperature signal. The long-term continuous measurement of the phase was conducted at constant temperatures of 30.5 °C, 30.60 °C, and 30.70 °C, as shown in Figure 4A.

The results shown in Figure 4A indicate that the temperature sensor based on the draw tower grating array has evident fluctuations in the measurement results at a constant temperature. The reason for these fluctuations was that the unbalanced Michelson interferometer used to compensate for the optical path difference was exposed to air, and the air temperature usually varies within ±1 °C. Therefore, we placed the unbalanced Michelson interferometer in the constant temperature state and remeasured the temperature. The measurement results are shown in Figure 4B, showing continuous measurements from 30.50 °C to 30.70 °C with an interval of 0.1 °C. The measurement time of the three constant temperatures was about 0.5 h. When the temperature changes from 30.50 °C to 30.60 °C, and then from 30.60 °C to 30.70 °C, the phase shows a continuous downward trend, then a steady rise, and finally tends to be stable. The reason for this phenomenon is the PID algorithm adopted by RTS-0515 [24]. When the thermostat enters the constant temperature state, there is an action to stop heating, which causes the temperature to show a trend of rising and then falling, thus resulting in the corresponding change in phase. The temperature fluctuation of the constant temperature equipment RTS used is ±0.001–0.01 °C, which indicates that the corresponding phase change should be approximately ±11.498–114.98 rad theoretically according to Equation (3). The inset in Figure 4B shows the result of continuous measurement under a constant temperature of 30.60 °C, where the phase changes in the range of −2010–−1900 rad agree well with the theoretical results. Therefore, in the actual constant temperature measurement process, the temperature changes slightly, which affects the phase of the signal. Additionally, it can be concluded from Figure 4B that when the temperature changes by 0.10 °C, the phase change was approximately 1000 rad. According to the fluctuation of the phase change in Figure 4A, the fluctuation range of room temperature was within ±1 °C, which agrees with the actual situation when the air condition is working.

**Figure 5:**
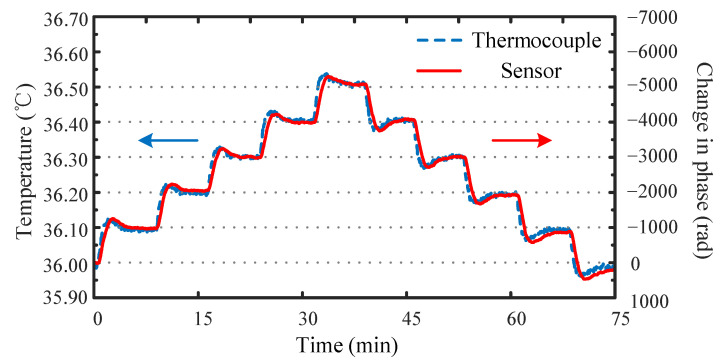
Heating and cooling response times of a sensor for the 36.00–36.50–36.00 °C loop.

The measurements were conducted for a thermal loop from 36.00 °C to 36.50 °C, and back to 36.00 °C, in steps of 0.10 °C. A thermocouple (DW1222) was used as a reference to record the change in actual temperature, whose measurement error is ±0.01 °C. The results for the thermal loop are shown in Figure 5.

Figure 5 illustrates the performance of the system in practical situations, where the red line indicates the changes in the phases which were applied while the temperature was continuously changing, and the blue line shows the temperature measured by the thermocouple when the water temperature changes constantly. The results shown in Figure 5 indicate a fair agreement between applied and measured values, thereby making evident the ability of the system to demodulate temperature signals that vary slowly in time. Generally, when the temperature drops or rises to a constant value, our temperature sensor responds more slowly than the reference thermocouple. This condition may be because our sensor is composed of an optical fiber wound around a cylindrical copper tube with a diameter of 6 cm and a height of 15 cm. The main purpose of protecting the sensor with a close-fitting copper tube was to keep the fibers tightly in the axial direction so that the measurements were strictly related to temperature and not affected by unwanted effects of bending or vibration. However, this approach makes our sensor much larger than the thermocouple sensor. The large size of our sensor causes it to need more time to sense temperature changes. Moreover, the phase change curve when the temperature rises was smoother than that when the temperature falls, because the constant temperature water tank turns on the refrigeration compressor when the temperature is lowered, thereby causing the water in the RTS-0515 to produce subtle vibrations. This vibration affects the change in the optical fiber phase, so the performance of the sensor is reduced when the temperature drops. However, both the rising and falling temperature stages are shown as symmetrical ladder shapes. The temperature changed by 0.10 °C under the thermal loop, and the corresponding phase changes were all approximately 1000 rad. This phenomenon, which is consistent with the measurement results in Figure 4, shows that our sensor has high stability in measuring temperature changes to achieve a high resolution of 0.10 °C.

**Figure 6:**
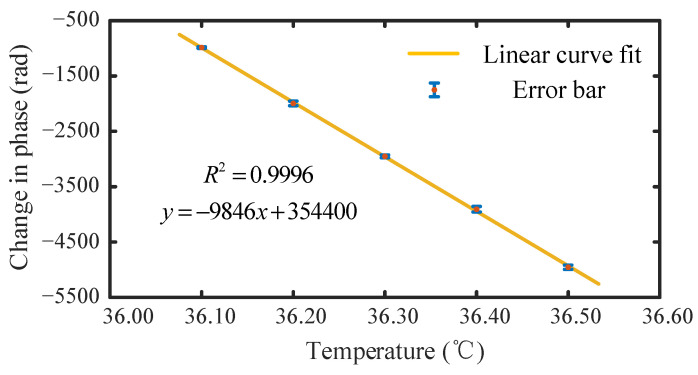
Fitted curve between phase and temperature.

A linear equation was fitted to the monitored and temperature data in a range of 36.10–36.50 °C, in steps of 0.10 °C, where the slope represents the temperature sensitivity factor of the sensor, the temperature sensitivity was 9846 rad/°C, and the Pearson squared correlation coefficient was $R^{2}=0.9996$, as outlined Figure 6.

**Figure 7:**
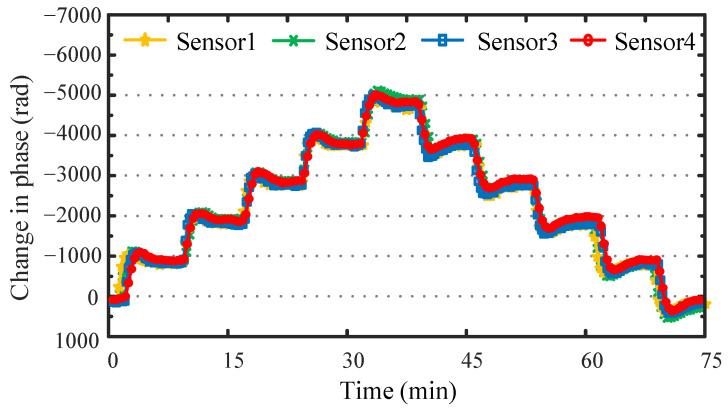
Demodulation experiment of temperature sensing array.

In this study, we constructed a temperature sensing array composed of four primitives, and tested the array in a loop temperature field from 35.00 °C to 35.50 °C, and back to 35.00 °C, in steps of 0.10 °C. The demodulation time domain results are shown in Figure 7; it can be found that there are slight differences in the demodulation amplitudes of the four primitives, caused by the different distribution positions of each primitive in the constant temperature water tank. However, it can be seen that overall, the time domain characteristics of the temperature signal obtained by the temperature sensor array under the same temperature field perform well.

**Figure 8:**
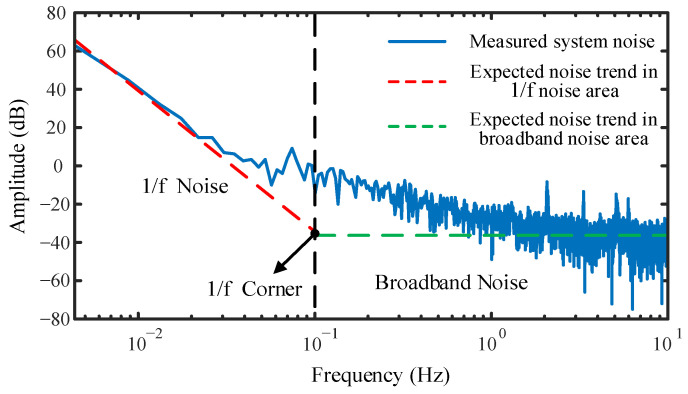
Background noise of fiber-optic temperature sensor.

In the experimental measurement process, owing to the limited accuracy of the constant temperature water tank equipment, only the experiment with a temperature resolution of 0.10 °C can be completed. To evaluate if our experimental device can achieve a temperature resolution higher than 0.10 °C, we measured the background noise of the system at a constant temperature of 35.00 °C, as shown in Figure 8. The left part in Figure 7 is the 1/f noise area, and the noise in this area mainly comes from the interaction between the system and the random forces of the external environment [25]. The right part is the broadband noise area, also known as white noise; the phase change of this area tends to be flat, and its average phase is −35.4 dB, which is equivalent to 0.017 rad. By analyzing the experimental results, Equation (3) shows that the order of magnitude of our system temperature resolution can reach 10^−5^ °C. Additionally, because the background noise is tested in an open environment, large-scale equipment such as incubators used in the experiment produce noise, which affects the system noise measurement. According to further analysis, placing the sensor in an anechoic chamber or a vacuum environment can obtain a lower background noise of the system.

## 4. Conclusions

In this paper, we proposed an FP interference structure based on the draw tower grating array for high-resolution temperature measurements. The two adjacent FBGs were used as mirrors and an optical fiber connecting them formed a sensor unit. The single beam was divided into reference light and probe light. The optical path difference between the two arms of the unbalanced Michelson interferometer was used to compensate for the optical path difference between the reference light and the probe light to detect the signal. The interaction between the sensor unit and the unbalanced Michelson interferometer created an interference peak that shifted linearly with temperature. The dual-beam common optical path design greatly reduced the complexity of the system. As the experimental setup only had the inconsistency of the light wave phase in the sensing part, the noise of the system was reduced, and the impact of polarization changed. The sensor was calibrated in the range of 36.00–36.50 °C, and back to 36.00 °C, in steps of 0.10 °C. A thermocouple (DW1222) was used as a reference. Experimental testing demonstrated that under the thermal loop, the temperature changed by 0.10 °C. Furthermore, the corresponding phase changes were all approximately 1000 rad. The results showed that the temperature and phase can be approximated as a linear relationship, and the Pearson square correlation coefficient was 0.9996. Furthermore, the temperature sensitivity was −9846 rad/°C, which showed that our temperature sensor had good measurement accuracy and stability as well as application prospects in ocean temperature measurement. To prove that our experimental device can achieve a higher temperature resolution, we measured the background noise of the system. The experimental results indicate that our system can achieve a high-resolution temperature measurement of the order of 10^−5^ °C. Owing to the favorable response, the temperature sensor array has advantages in ocean environment monitoring applications. Moreover, because the sensor was based on the draw tower grating array, our temperature sensor system demonstrated notable potential in large-scale multiplexing.
